# Pediatric candidemia epidemiology and antifungal susceptibility profiles reported by the ISPED program in China, 2016–2024

**DOI:** 10.1128/aac.00031-26

**Published:** 2026-06-02

**Authors:** Jun Xu, Pan Fu, Hong-Mei Xu, Chun-Mei Jing, Ji-Kui Deng, Hong-Mei Wang, Chun-Zhen Hua, Ying-Hu Chen, Xue-Jun Chen, Ming-Ming Zhou, Ting Zhang, Hong Zhang, Yi-Ping Chen, Jin-Hong Yang, Ji-An Li, Ai-Wei Lin, Shi-Fu Wang, Qing Cao, Xing Wang, Hui-Ling Deng, Hui-Jun Cai, San-Cheng Cao, Jian-Hua Hao, Wei Gao, Yu-Yang Zhou, Yuan-Yuan Huang, Zhi-Qiang Zhuo, Mei-Lian Huang, Hong-Xia Xiang, Chuan-Qing Wang, Hui Yu

**Affiliations:** 1 Infectious Disease Department, Children’s Hospital of Fudan University, National Children's Medical Center, Shanghai, China; 2 Clinical Microbiology Laboratory Department, Children’s Hospital of Fudan University, National Children's Medical Center, Shanghai, China; 3 Infectious Disease Department, Children's Hospital of Chongqing Medical University 159456, Chongqing, China; 4 Clinical Microbiology Laboratory Department, Children's Hospital of Chongqing Medical University 159456, Chongqing, China; 5 Infectious Disease Department, Shenzhen Children’s Hospital, Guangzhou, China; 6 Infectious Disease Department, Zhejiang University School of Medicine Children's Hospital 605254 , Hangzhou, Zhejiang, China; 7 Clinical Microbiology Laboratory Department, Zhejiang University School of Medicine Children's Hospital 605254 , Hangzhou, Zhejiang, China; 8 Infectious Disease Department, Shanghai Children’s Hospital (affiliated with Shanghai Jiao tong University School of Medicine) 36777, Shanghai, China; 9 Clinical Microbiology Laboratory Department, Shanghai Children’s Hospital (affiliated with Shanghai Jiao tong University School of Medicine) 36777, Shanghai, China; 10 Pediatric Infectious Disease Department, The Second Affiliated Hospital and Yuying Children's Hospital of Wenzhou Medical University 26452, Wenzhou, Zhejiang, China; 11 Clinical Microbiology Laboratory Department, The Second Affiliated Hospital and Yuying Children's Hospital of Wenzhou Medical University 26452, Wenzhou, Zhejiang, China; 12 Infectious Disease Department, Jinan Children’s Hospital (Children’s Hospital Affiliated Shandong University), Jinan, Shandong, China; 13 Clinical Microbiology Laboratory Department, Jinan Children’s Hospital (Children’s Hospital Affiliated Shandong University), Jinan, Shandong, China; 14 Infectious Disease Department, Shanghai Children’s Medical Center (affiliated with Shanghai Jiao tong University School of Medicine) 56694, Shanghai, China; 15 Clinical Microbiology Laboratory Department, Shanghai Children’s Medical Center (affiliated with Shanghai Jiao tong University School of Medicine) 56694, Shanghai, China; 16 Infectious Disease Department, Xi’an Jiao tong University Affiliated Children’s Hospital 12480, Xi'an, Shaanxi, China; 17 Clinical Microbiology Laboratory Department, Xi’an Jiao tong University Affiliated Children’s Hospital, Xi’an, Shaanxi, China; 18 Infectious Disease Department, Children’s Hospital of Kaifeng, Kaifeng, Henan, China; 19 Clinical Microbiology Laboratory Department, Children’s Hospital of Kaifeng, Kaifeng, Henan, China; 20 Pediatrics, Bethune First Hospital of Jilin University, Changchun, Jilin, China; 21 Infectious Disease Department, Xiamen Children's Hospital (Children's Hospital of Fudan University at Xiamen) 669385, Xiamen, Fujian, China; 22 Clinical Microbiology Laboratory Department, Xiamen Children's Hospital (Children's Hospital of Fudan University at Xiamen) 669385, Xiamen, Fujian, China; 23 Infectious Disease Department, Wuxi Children’s Hospital, Wuxi, Jiangsu, China; University Children's Hospital Münster, Münster, Germany

**Keywords:** candidemia, epidemiology, antifungal susceptibility, non-*albicans Candida*

## Abstract

Candidemia is life-threatening in children. Limited epidemiological and antifungal resistance data for children necessitate national surveillance. This multicenter retrospective study (2016–2024) analyzed 527 pediatric candidemia cases from 13 children’s hospitals via the Infectious Disease Surveillance of Pediatrics (ISPED) network. *Candida* species caused 96.0% (527/549) of fungemia cases. Neonates and infants comprised 51.6% of cases (24.5% neonates and 27.1% infants). Non*-albicans Candida* species (NACs) dominated (74.0%), with the prevalence of *Candida parapsilosis* increasing from 21.2% in 2019 to 51.9% in 2024 (*Z* = 3.11, *P* = 0.002), while the prevalence of *C. albicans* decreased from 38.9% to 15.6% during the same period (*Z* = −2.25, *P* = 0.024). Azole susceptibility of NACs decreased significantly: *C. parapsilosis* fluconazole susceptibility decreased from 93.5% (2016–2018) to 63.6% (2022–2024) (*P* < 0.001), whereas voriconazole (79.5%–93.3%, *P* = 0.078) and itraconazole (80.0%–97.4%, *P* = 0.369) susceptibility remained high. *Candida tropicalis* showed low azole susceptibility, with significant decreases in susceptibility to fluconazole (61.5% in 2016–2018 to 35.0% in 2022–2024) and voriconazole (61.5% in 2016–2018 to 20.0% in 2022–2024; *P* < 0.001 for all). The susceptibility of wild-type *Candida guilliermondii* decreased toward fluconazole (100.0% in 2016–2018 and 2019–2021 to 38.9% in 2022–2024), voriconazole (100.0% in 2016–2018 and 2019–2021 to 27.8% in 2022–2024), and itraconazole (100.0% in 2016–2018 and 2019–2021 to 22.2% in 2022–2024) (*P* < 0.001 for all). All *Candida* species showed high susceptibility to amphotericin B and echinocandins (99.0% and 95.9%, respectively). Overall, there was a notable shift toward NACs, especially *C. parapsilosis*, *C. tropicalis*, and *C. guilliermondii*, accompanied by declining susceptibility to azoles (particularly in *C. tropicalis* and *C. guilliermondii*), which underscores the urgent need for enhanced antifungal stewardship and continuous resistance monitoring.

## INTRODUCTION

Candidemia is among the most frequent causes of life-threatening infections and poses significant clinical challenges because of its high morbidity and mortality. In October 2022, *Candida* species were classified as a “critical priority” in the Fungal Priority Pathogen List (FPPL) by the World Health Organization (WHO) (1). This document highlights the significant public health threat posed by fungal infections and the growing concern regarding antifungal resistance (AFR) among key pathogens (2).

Fungi, especially *Candida* species, are frequently isolated from bloodstream infections (BSIs), whose prevalence has increased in recent decades (3). In 2017, it was estimated that *Candida* species were responsible for 10.0% of BSIs in Canada (4). Data from the China Antimicrobial Surveillance Network (CHINET) in 2016 indicated that fungi accounted for 2.9% of BSI isolates (5). More importantly, *Candida* species exhibit significant diversity and adaptability, with increasing drug resistance being monitored globally, and the emergence of AFR strains contributes to the global health burden (3, 6).

The pathogenic spectrum of candidemia is undergoing a global shift from *C. albican*s toward non*-albicans Candida* species (NACs), yet with marked regional variations. While *C. albicans* remains predominant in parts of Europe and Asia, NACs such as *Candida parapsilosis* and *Candida tropicalis* are becoming more prevalent in North America, southern Europe, and certain Asian regions (7–11). This shift may be associated with widespread azole prophylactic and therapeutic use, presenting significant challenges for clinical management because of the distinct AFR profiles exhibited by NACs (2, 12).

Pediatric patients are at a high risk for candidemia because of their immature immune systems and health issues, leading to different epidemiological and AFR patterns than those of adults (13). However, current studies have limited sample sizes and provided insufficient data in the pediatric population. Therefore, national surveillance programs are urgently needed, and AFR profiles derived from such data are essential for enhancing pediatric patient outcomes and reducing substantial health care costs. The Infectious Disease Surveillance of Pediatrics (ISPED) program was established in 2015 to monitor pathogenic microorganisms and antimicrobial resistance. Currently, it encompasses 13 tertiary care children’s hospitals across nine major Chinese provinces and autonomous regions. Here, we present the first comprehensive 9-year analysis of candidemia epidemiology and AFR patterns in Chinese children based on the data from the ISPED network.

## RESULTS

### Demographic characteristics and species distribution

**Fig 1:**
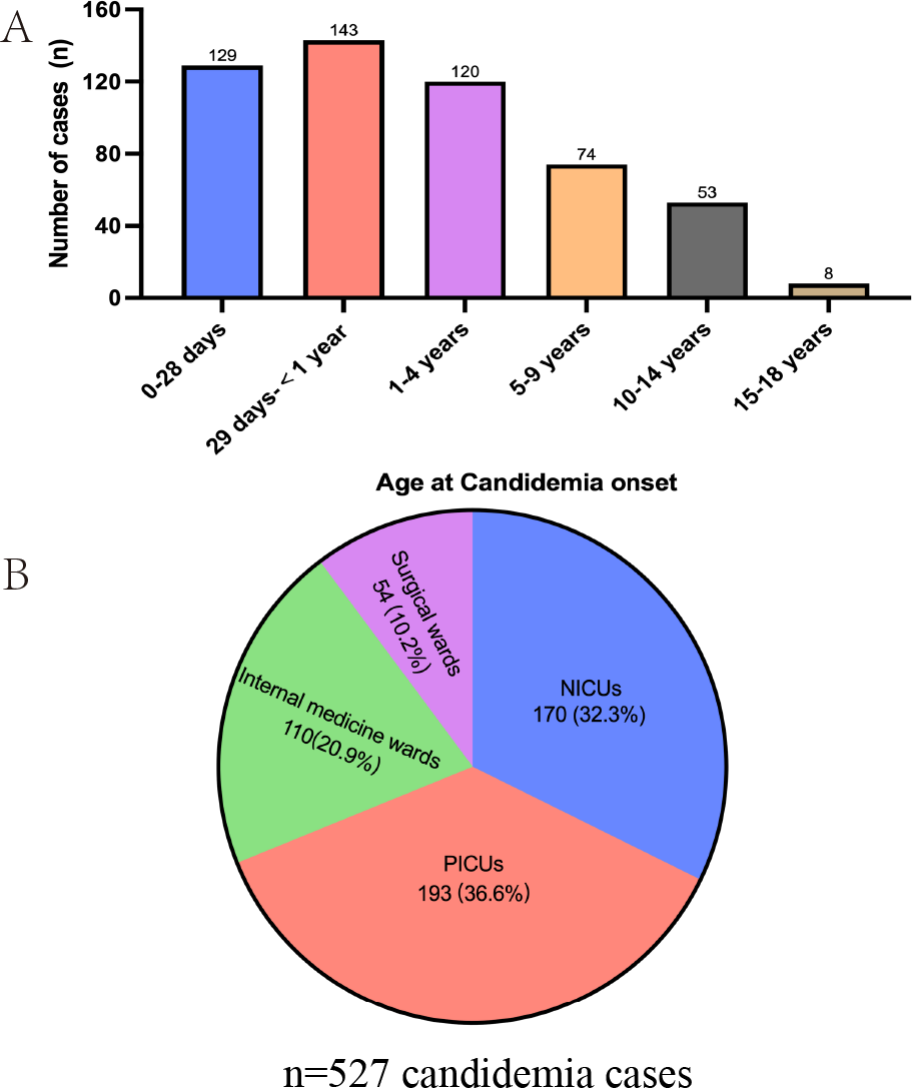
Demographic characteristics of pediatric candidemia cases (*n* = 527candidemia cases). (**A**) Age distribution of pediatric candidemia cases. (**B**) Ward-type distribution of pediatric candidemia cases.

This multicenter analysis collected 618 fungal bloodstream isolates from January 2016 to December 2024. Among these, 69 isolates were excluded: 66 because of repeated isolates from the same cases, two because of incomplete records, and one because of difficulties in species identification. Overall, 549 cases of fungemia were included. *Candida* species accounted for 96.0% (*n* = 527) of fungemia, and rare pathogens included *Aspergillus* species (5, 0.9%), *Cryptococcus neoformans* (4, 0.7%), and other fungi (13, 2.4%). In total, 527 candidemia cases were collected. Notably, more than half of candidemia cases (272, 51.6%) were recorded in neonates (129, 24.5%) and infants (143, 27.1%; Fig. 1A). The highest burden was recorded in pediatric intensive care units (PICUs; 193, 36.6%), followed by neonatal intensive care units (NICUs; *n* = 170, 32.3%), internal medicine wards (110, 20.9%), and surgical wards (54, 10.2%; Fig. 1B).

### Temporal shift in species of pediatric candidemia

**Fig 2:**
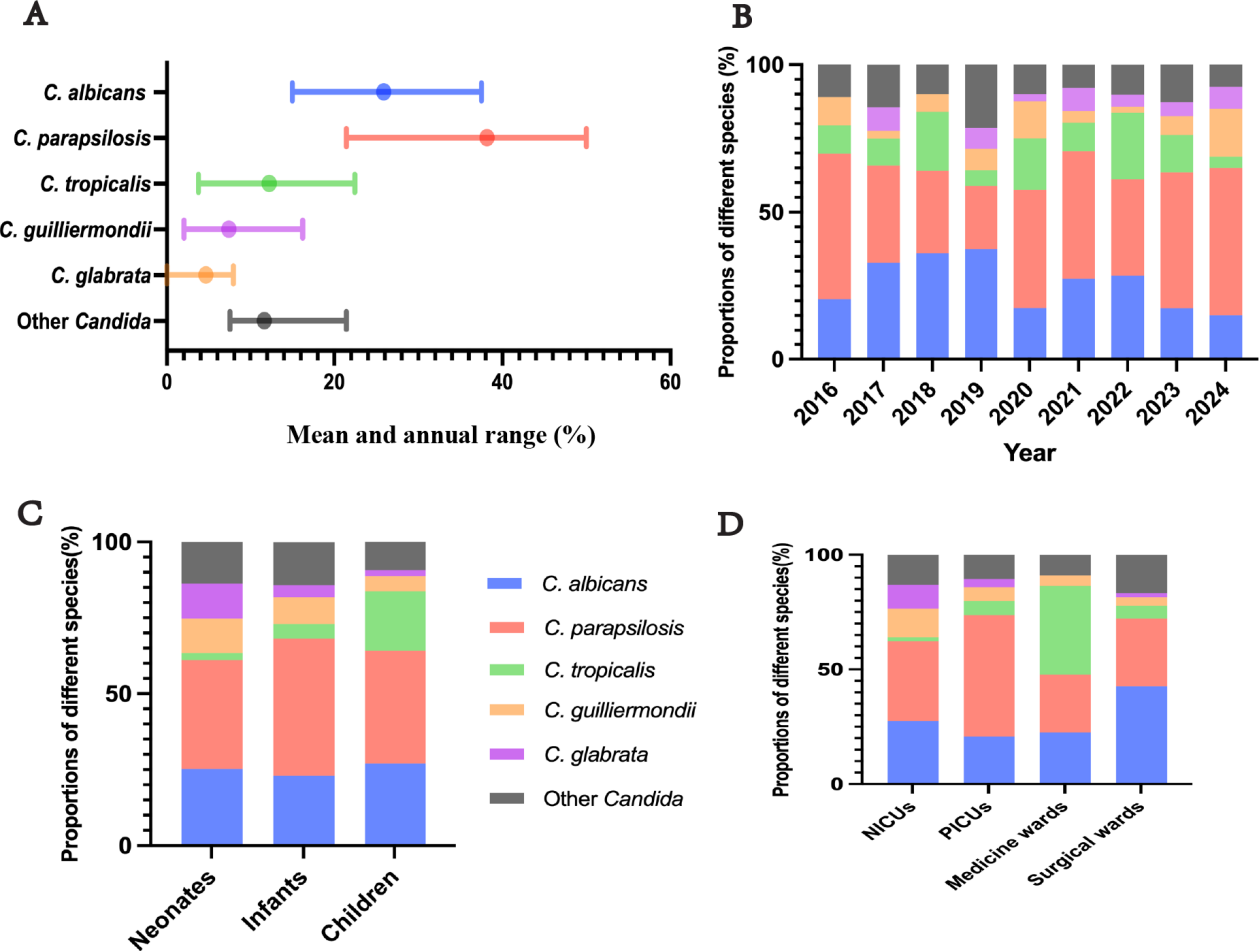
*Candida* species distribution in pediatric candidemia cases from 2016 to 2024. (**A**) *Candida* species proportions in pediatric candidemia (mean and annual range). (**B**) Temporal species distribution of pediatric candidemia. (**C**) Species distribution of candidemia in different pediatric populations. (**D**) Ward-specific distribution of pediatric candidemia.

Over the 9-year period (2016–2024), *C. albicans* was not the most frequently isolated species in pediatric candidemia. It accounted for only 26.0% (range: 15.6%–38.9% per year) of the 527 cases, was initially predominant (peaking at 38.9% in 2019), and declined sharply to 15.6% by 2024 (Cochran–Armitage *Z* = −2.25, *P* = 0.024). In contrast, NACs were responsible for a significant proportion, comprising 74.0% of the cases (range: 61.1%–84.4% per year). Among these, *C. parapsilosis* emerged as the most consistently dominant species (39.8%, range: 22.2%–51.9% per year) and exhibited a strong, statistically significant upward trend (Cochran-Armitage *Z* = 3.11, *P* = 0.002). Other NACs included *C. tropicalis* (11.6%), *Candida guilliermondii* (7.8%), and *Candida glabrata* (4.9%). A notable surge was observed in *C. guilliermondii* cases, which increased from <10.00% in prior years to 16.9% in 2024 (Monte Carlo *P* = 0.019). *C. tropicalis* fluctuated annually (peak: 23.9% in 2022; decline: 3.9% in 2024), while *C. glabrata cases* remained sporadic (0%–7.9%; Fig. 2A and B). Additionally, only two *Candida krusei* cases were detected during this period.

### Age- and ward-specific distributions

*C. glabrata* was more frequent in neonates (15/129, 11.6%) than in infants (6/143, 4.2%) or children (5/255, 2.0%; Fisher’s exact *P* < 0.001). However, compared with its prevalence in neonates (3/129, 2.3%) and infants (7/143, 4.9%), *C. tropicalis* predominated in children (20.0%, 51/255; Fisher’s exact *P* < 0.001). No significant differences were detected in the proportions of *C. albicans*, *C. parapsilosis*, *C. guilliermondii*, and other *Candida* species across age groups (*χ*^2^ = 0.81, 4.78, 5.59, and 0.54; all *P* > 0.05; Fig. 2C). A significant association was identified between different wards and *Candida* species distribution (*χ*² = 114.52, *P* < 0.001). Distinct species predominance patterns emerged across wards: *C. parapsilosis* dominated NICUs (35.9%, 61/170) and PICUs (54.4%, 105/193), while *C. tropicalis* was most prevalent in internal medicine wards (39.1%, 43/110). Surgical wards exhibited a high prevalence of *C. albicans (*42.6%, 23/54; Fig. 2D).

### *Candida* species susceptibility in pediatric candidemia

The MICs of antifungal agents against 410 *Candida* isolates are summarized in Table 1. Fluconazole exhibited relatively high activity against *C. albicans* (86.2% MIC ≤ 2 µg/mL), *C. parapsilosis* (77.6% MIC ≤ 2 µg/mL), and *C. glabrata* (95.5% MIC ≤ 32 µg/mL) but had limited efficacy against *C. tropicalis* (44.2% MIC ≤ 2 µg/mL). In total, 48.2% of the *C. guilliermondii* isolates were exhibiting a MIC > 16 µg/mL.

**TABLE 1:** Susceptibility of *Candida* isolates in the pediatric population^*a*^

Antifungal and *Candida* sp.	Cases (*n*)	No. (%) of isolates with MIC value (μg/mL) of:						
		**≤2**	**4**	**8**	**16**	**32**	**64**	**≥128**
FLU								
*C. albicans*	109	94 (86.2)	3 (2.8)	3 (2.8)	5 (4.6)	1 (0.9)	1 (0.9)	2 (1.8)
*C. parapsilosis*	161	125 (77.6)	11 (6.8)	9 (5.6)	7 (4.3)	4 (2.5)	3 (1.9)	2 (1.3)
*C. tropicalis*	43	19 (44.2)	1 (2.3)	2 (4.7)	3 (7.0)	1 (2.3)	2 (4.7)	15 (34.8)
*C. glabrata*	22	16 (72.8)	5 (22.7)	0	0	0	1 (4.5)	0
*C. guilliermondii*	27	9 (33.3)	3 (11.1)	2 (7.4)	0	0	1 (3.7)	12 (44.5)
*Candida lusitaniae*	10	10 (100.0)	0	0	0	0	0	0
Other	31	16 (51.5)	10 (32.3)	2 (6.5)	2 (6.5)	0	0	1 (3.2)
		**No. (%) of isolates with MIC value (μg/mL) of:**						
		**≤0.125**	**0.25**	**0.5**	**1**	**2**	**4**	**≥8**
VOR								
*C. albicans*	109	78 (71.6)	11 (10.1)	11 (10.1)	5 (4.6)	1 (0.9)	1 (0.9)	2 (1.8)
*C. parapsilosis*	161	138 (85.7)	7 (4.4)	1 (0.6)	6 (3.7)	4 (2.5)	1 (0.6)	4 (2.5)
*C. tropicalis*	43	16 (37.2)	4 (9.3)	1 (2.3)	2 (4.7)	3 (7.0)	1 (2.3)	16 (37.2)
*C. glabrata*	14	10 (71.5)	2 (14.3)	0	1 (7.1)	0	0	1 (7.1)
*C. krusei*	2	0	1 (50.0)	0	1 (50.0)	0	0	0
*C. guilliermondii*	27	14 (51.9)	0	0	1 (3.7)	0	1 (3.7)	11 (40.7)
*C. lusitaniae*	10	9 (90.0)	1 (10.0)	0	0	0	0	0
Other	31	18 (58.1)	1 (3.2)	9 (29.0)	2 (6.5)	1 (3.2)	0	0
		**No. (%) of isolates with MIC value (μg/mL) of:**						
		**≤0.125**	**0.25**	**0.5**	**1**	**2**	**4**	**≥8**
ITR								
*C. albicans*	81	67 (82.7)	6 (7.4)	2 (2.4)	3 (3.7)	0	1 (1.2)	2 (2.6)
*C. parapsilosis*	159	139 (87.5)	14 (8.8)	1 (0.6)	2 (1.3)	1 (0.6)	1 (0.6)	1 (0.6)
*C. tropicalis*	37	8 (21.7)	4 (10.8)	4 (10.8)	5 (13.5)	7 (18.9)	6 (16.2)	3 (8.1)
*C. glabrata*	21	10 (47.6)	2 (9.5)	3 (14.3)	4 (19.0)	1 (4.8)	1 (4.8)	0
*C. guilliermondii*	27	12 (44.5)	1 (3.7)	5 (18.5)	0	1 (3.7)	1 (3.7)	7 (25.9)
*C. lusitaniae*	12	12 (100.0)	0	0	0	0	0	0
Other	31	21 (67.8)	5 (16.1)	3 (9.7)	1 (3.2)	1 (3.2)	0	0
		**No. (%) of isolates with MIC value (μg/mL) of:**						
		**≤0.125**	**0.25**	**0.5**	**1**	**2**	**4**	**≥8**
AMB								
*C. albicans*	100	5 (5.0)	52 (52.0)	41 (41.0)	2 (2.0)	0	0	0
*C. parapsilosis*	161	83 (51.5)	18 (11.2)	47 (29.2)	13 (8.1)	0	0	0
*C. tropicalis*	42	0	4 (9.5)	25 (59.5)	11 (26.2)	2 (4.8)	0	0
*C. glabrata*	23	1 (4.4)	2 (8.7)	16 (69.6)	3 (13.0)	1 (4.3)	0	0
*C. krusei*	2	0	0	1 (50.0)	1 (50.0)	0	0	0
*C. guilliermondii*	27	5 (18.5)	7 (25.9)	14 (51.9)	1 (3.7)	0	0	0
*C. lusitaniae*	10	4 (40.0)	2 (20.0)	4 (40.0)	0	0	0	0
Other	31	4 (12.9)	0	24 (77.4)	2 (6.5)	0	1 (3.2)	0
		**No. (%) of isolates with MIC value (μg/mL) of:**						
		**≤4**	**8**	**16**	**32**	**64**	**≥128**	**/**
FCT								
*C. albicans*	96	93 (97.0)	0	0	1 (1.0)	1 (1.0)	1 (1.0)	/
*C. parapsilosis*)	157	153 (97.4)	0	0	0	2 (1.3)	2 (1.3)	/
*C. tropicalis*	42	41 (97.6)	1 (2.4)	0	0	0	0	/
*C. glabrata*	20	20 (100.0)	0	0	0	0	0	/
*C. krusei*	2	2 (100.0)	0	0	0	0	0	
*C. guilliermondii*	27	15 (55.6)	0	0	0	7 (25.9)	5 (18.5)	/
*C. lusitaniae*	12	12 (100.0)	0	0	0	0	0	/
Other	31	30 (96.8)	0	1 (3.2)	0	0	0	/
		**No. (%) of isolates with MIC value (μg/mL) of:**						
		**≤0.125**	**0.25**	**0.5**	**1**	**2**	**4**	**≥8**
Micafungin								
*C. albicans*	11	10 (90.9)	1 (9.1)	0	0	0	0	0
*C. parapsilosis*	20	5 (25.0)	3 (15.0)	6 (30.0)	6 (30.0)	0	0	0
*C. tropicalis*	8	5 (62.5)	2 (25.0)	0	0	0	0	1 (12.5)
*C. glabrata*	2	2 (100.0)	0	0	0	0	0	0
*C. guilliermondii*	4	2 (50.0)	0	1 (25.0)	0	0	0	1 (25.0)
*C. lusitaniae*	2	2 (100.0)	0	0	0	0	0	0
Other	2	2 (100.0)	0					0
		**No. (%) of isolates with MIC value (μg/mL) of:**						
		**≤0.125**	**0.25**	**0.5**	**1**	**2**	**4**	**≥8**
Caspofungin								
*C. albicans*	11	11 (100.0)	0	0	0	0	0	0
*C. parapsilosis*	20	2 (10.0)	2 (10.0)	4 (20.0)	12 (60.0)	0	0	0
*C. tropicalis*	8	7 (87.5)	0	0	0	1 (12.5)	0	0
*C. glabrata*	2	2 (100.0)	0	0	0	0	0	0
*C. guilliermondii*	4	1 (25.0)	2 (50.0)	1 (25.0)	0	0	0	0
*C. lusitaniae*	2	2 (100.0)	0	0	0	0	0	0
Other	2	2 (100.0)	0	0	0	0	0	0
		**No. (%) of isolates with MIC value (μg/mL) of:**						
		**≤0.125**	**0.25**	**0.5**	**1**	**2**	**4**	**≥8**
Anidulafungin								
*C. albicans*	11	10 (90.9)	1 (9.1)	0	0	0	0	0
*C. parapsilosis*	20	3 (15.0)	0	3 (15.0)	12 (60.0)	2 (10.0)	0	0
*C. tropicalis*	8	5 (62.5)	2 (25.0)	0	0	1 (12.5)	0	0
*C. glabrata*	2	2 (100.0)	0	0	0	0	0	0
*C. guilliermondii*	4	1 (25.0)	0	1 (25.0)	1 (25.0)	1 (25.0)	0	0
*C. lusitaniae*	2	2 (100.0)	0	0	0	0	0	0
Other	2	2 (100.0)	0	0	0	0	0	0

^*a*^ FCT, flucytosine; VOR, voriconazole; ITR, itraconazole; AMB, amphotericin B; FLC, fluconazole.

Voriconazole and itraconazole potently inhibited *C. albicans* (MIC ≤ 0.125 µg/mL in 71.6% and 82.7% of isolates, respectively), *C. parapsilosis* (MIC ≤ 0.125 µg/mL in 85.7% and 87.4% of isolates, respectively), and *C. glabrata* (MIC ≤ 0.5 µg/mL in 85.7% of isolates and MIC ≤ 0.25 µg/mL in 57.1% of isolates). In contrast, the MICs of voriconazole and itraconazole were ≤0.125 µg/mL against only 37.2% and 21.6% of *C. tropicalis* isolates, whereas the MIC was ≤0.125 µg/mL against 51.9% and 44.5% of *C. guilliermondii* isolates, respectively.

Amphotericin B (AMB) showed broad-spectrum activity (99.0% susceptibility at MIC ≤ 1 µg/mL), except against two *C. tropicalis* isolates, *C. glabrata* isolates, and one *C. haemulonii* isolate. However, *C. tropicalis* isolates required higher AMB concentrations (26.2%; MIC = 1 µg/mL) than *C. albicans* (2.0%; Fisher’s exact *P* < 0.001) and *C. parapsilosis* (8.1%; *χ^2^* = 8.40, *P* = 0.004).

Flucytosine was highly effective against most species, with susceptibility rates ranging from 97.1% to 100.0% for *C. albicans*, *C. parapsilosis*, *C. tropicalis*, *C. glabrata*, and others. Strikingly, 44.5% of *C. guilliermondii* isolates displayed resistance to flucytosine (MIC ≥ 32 µg/mL).

Moreover, susceptibility of 49 *Candida* isolates, including *C. albicans* (*n* = 11), *C. parapsilosis* (*n* = 20), *C. tropicalis* (*n* = 8), *C. glabrata* (*n* = 2), *C. guilliermondii* (*n* = 4), and other *Candida* species (*n* = 4), to echinocandins was determined. A total of 95.9% (47/49) of these isolates were sensitive to all echinocandins, except for one *C. tropicalis* isolate. One *C. guilliermondii* isolate was resistant to micafungin but susceptible to caspofungin/anidulafungin.

### Azole resistance trends in pediatric candidemia

**Fig 3:**
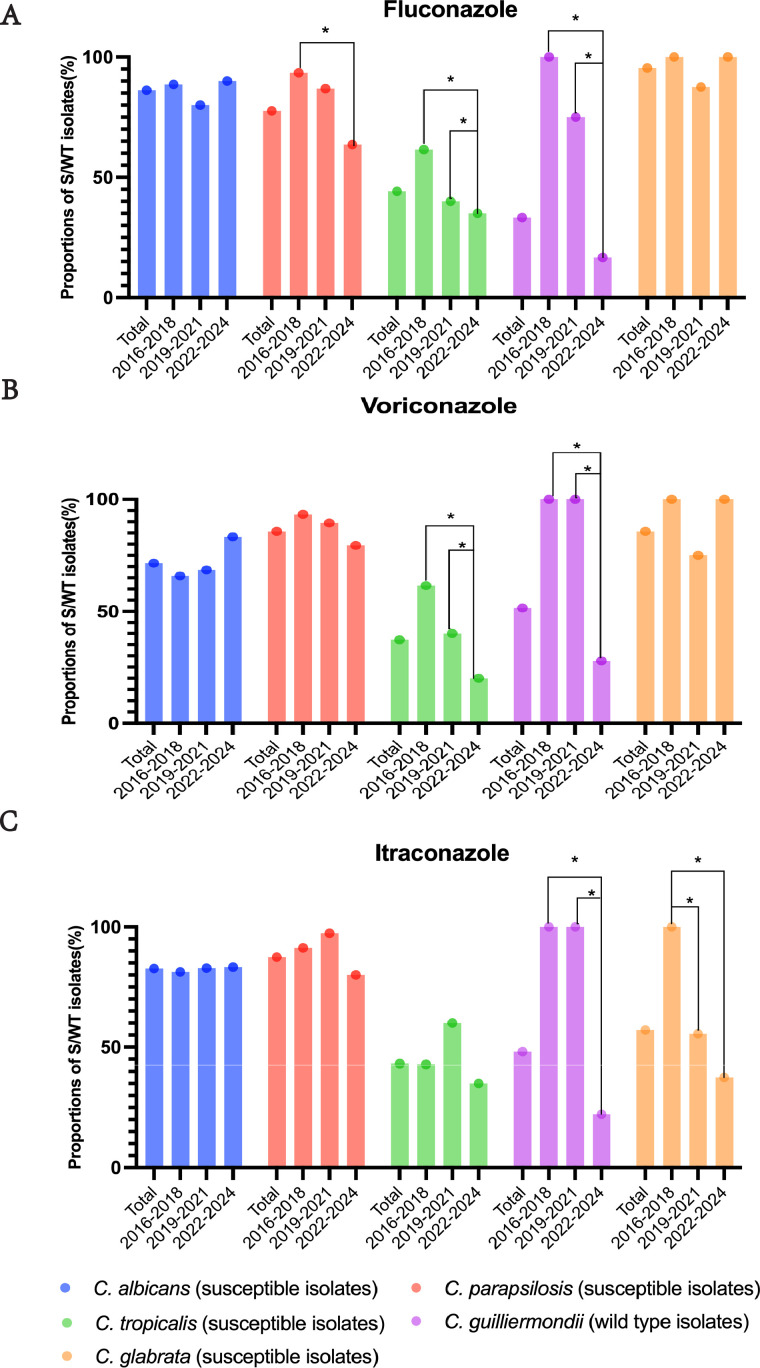
Antifungal susceptibility of *Candida* species in pediatric candidemia. (**A**) *Candida* species susceptibility/epidemiological cutoff value (ECV) to fluconazole in pediatric candidemia. (**B**) *Candida* species susceptibility/ECV to voriconazole in pediatric candidemia. (**C**) *Candida* species susceptibility/ECV to itraconazole in pediatric candidemia. S, susceptible; WT, wild type. For species with established CLSI or EUCAST clinical breakpoints, isolates were categorized as susceptible (S). For species lacking CLSI/EUCAST-defined breakpoints, ECVs were used to categorize isolates as WT. Wild-type isolates = susceptible isolates. * *P* < 0.05.

Data from 2016 to 2024 revealed significant temporal shifts in antifungal susceptibility rates among *Candida* isolates. Antifungal susceptibility differs across different species. This study evaluated the susceptibility trends of five *Candida* species to fluconazole, voriconazole, and itraconazole across three periods (2016–2018, 2019–2021, and 2022–2024). NACs exhibited a significant decrease in susceptibility to azoles. The susceptibility of *C. parapsilosis* to fluconazole decreased from 93.5% (2016–2018) to 63.6% (2022–2024; Fisher’s exact *P*<0.001), but susceptibility to voriconazole (79.5%–93.3%; Fisher’s exact *P* = 0.078) and itraconazole (80.0%–97.4%; Fisher’s exact *P* = 0.369) remained stable. *C. tropicalis* exhibited low susceptibility to all azoles (fluconazole 44.2%, voriconazole 37.2%, and itraconazole 43.2%), with significant decreases in susceptibility to fluconazole (61.5% in 2016–2018 to 35.0% in 2022–2024; Fisher’s exact *P* < 0.001) and voriconazole (61.5% in 2016–2018 to 20.0% in 2022–2024; Fisher’s exact *P* < 0.001), but not itraconazole (Fisher’s exact *P* = 0.056). The proportion of wild type (WT) *C. guilliermondii* plummeted for fluconazole (100.0% in 2016–2018 and 2019–2021 to 38.9% in 2022–2024; Fisher’s exact *P* < 0.001), voriconazole (100.0% in 2016–2018 and 2019–2021 to 27.8% in 2022–2024; Fisher’s exact *P* < 0.001), and itraconazole (100.0% in 2016–2018 and 2019–2021 to 22.2% in 2022–2024; Fisher’s exact *P* < 0.001). *C. glabrata* maintained high susceptibility to fluconazole (95.5%, range 87.5%–100.0%), voriconazole (85.7%, range 75.0%–100.0%), and itraconazole (57.1%, range 37.5%–100.0%), with no temporal variation toward fluconazole and voriconazole (Fisher’s exact *P* > 0.05), but susceptibility to itraconazole decreased (Fisher’s exact *P* = 0.001; Fig. 3). In contrast, *C. albicans* maintained relatively high overall susceptibility to fluconazole (86.2%, range 80.0%–90.0%), voriconazole (71.6%, range 65.9%–83.3%), and itraconazole (82.7%, range 81.3%–83.3%).

## DISCUSSION

Candidemia is a major cause of life-threatening infections and presents substantial clinical challenges because of its high morbidity and mortality (6, 13). To our knowledge, this study presents the largest investigation of pediatric candidemia in recent decades, providing a 9-year analysis (2016–2024) of epidemiology and AFR profiles across 13 children’s hospitals in China. Our findings indicated that *Candida* species (96.0%) are predominant in pediatric fungemia in China, with rare non-*Candida* fungi (4.0%). Crucially, we documented a shift from *C. albicans* dominance: NACs now constitute 74.0% of pediatric candidemia cases, with *C. parapsilosis* emerging as the leading pathogen. This finding contrasted with those of earlier Chinese studies (2011–2021), where *C. albicans* predominated (54.4%) among 68 pediatric patients, aligning more closely with global trends observed in Turkey (56.3% *C. parapsilosis*; 2012–2018) and northern Greece (33.6% *C. parapsilosis*; 2020–2024) (7, 14, 15). The increase in *C. parapsilosis* might reflect biofilm formation on medical devices and extensive azole prophylaxis (16), underscoring its adaptability in high-risk settings such as NICUs and PICUs. Concurrently, *C. guilliermondii* cases increased to 16.9% by 2024, corroborating Turkish data indicating that it is the third most common BSI yeast (17). *C. tropicalis* is prevalent in internal medicine wards and associated with immunocompromised hosts, exhibiting marked volatility (23.9% in 2022 vs. 3.9% in 2024) (18). These species pose distinct therapeutic challenges because of higher MICs or reduced azole susceptibility.

Notably, neonates and infants comprised 51.6% of the cases, reflecting heightened susceptibility, which was attributable to immature immunity and frequent invasive interventions (19). Age-specific disparities emerged: neonates exhibited higher *C. glabrata* prevalence than children did, necessitating the consideration of echinocandins, given this species’ reduced azole susceptibility. Ward-level epidemiology further informs risk stratification: PICUs and NICUs were dominated by *C. parapsilosis*, demanding enhanced catheter hygiene protocols and targeted antifungal therapies, while internal medicine wards showed *C. tropicalis* predominance, warranting rapid susceptibility testing in neutropenic patients (18, 20).

The evolving pathogen spectrum has introduced novel clinical challenges because of unique AFR profiles (2, 6). Alarmingly, susceptibility to azoles had dramatically decreased, driven primarily by NACs. The susceptibility of *C. parapsilosis* to fluconazole decreased significantly (93.5% in 2016–2018 to 63.6% in 2022–2024), but remained higher than that reported in northern Greece (52.0% resistance) (16). The susceptibility to voriconazole (79.5%–93.3%) and itraconazole (80.0%–97.4%) was relatively high.

The resistance of other NACs was more severe. *C. tropicalis* exhibited low susceptibility to all azoles, with significant decreases in susceptibility to fluconazole (61.5% in 2016–2018 to 35.0% in 2022–2024) and voriconazole (61.5% in 2016–2018 to 20.0% in 2022–2024). The susceptibility of WT *C. guilliermondii* also decreased across all azoles. Geographic heterogeneity in resistance rates exists; for example, on the southern Chinese island of Hainan, extensive genetic diversity and multiple origins of fluconazole resistance have been documented, suggesting that resistance emerges independently across strains (21, 22). This variation was potentially attributable to regional differences in antifungal stewardship or genetic clade divergence (15, 23).

Despite azole resistance concerns, AMB (99.0% susceptibility) and flucytosine (>95.0% susceptibility for most species) retained efficacy. However, flucytosine failed against 44.5% of *C. guilliermondii*. Echinocandins showed high activity among the tested isolates (95.9%); however, given that susceptibility testing was limited to a subset of isolates, these results should be interpreted cautiously and may not be generalizable across all centers.

The ISPED data reflected a convergence of NACs dominance and rising azole resistance globally (14, 24, 25). To mitigate treatment failure, comprehensive strategies are essential: restricting azole prophylaxis to low-risk patients, prioritizing echinocandins/AMB in NAC-prevalent settings, implementing rapid diagnostics for *C. tropicalis* resistance markers (e.g., ERG11 mutations), enforcing strict device audits and barrier precautions in NICUs/PICUs, and considering prophylactic AMB for high-risk preterm infants (26–28). Although no *C. auris* was detected in this study, global coordination of pediatric fungemia registries is crucial for monitoring emerging threats (15).

Limitations included the retrospective design (potential selection bias), restriction to 13 tertiary pediatric centers (limiting generalizability), and the absence of clinical outcome correlations. We employed Clinical and Laboratory Standards Institute (CLSI) methods primarily but adopted European Committee on Antimicrobial Susceptibility Testing (EUCAST) breakpoints for AMB due to the absence of CLSI equivalents, which may hinder comparability with studies utilizing a single standard. Future standardization efforts will be crucial for enhancing data consistency. Additionally, the lack of uniform echinocandin susceptibility testing across centers and varying local epidemiology could lead to underdetection of resistance. Future studies focusing on multicenter standardized surveillance are needed. Regrettably, we were unable to provide the incidence data for all centers. Systematic data will be collected in our future work.

In conclusion, *Candida* species caused nearly all pediatric fungemia (96.0%), predominantly affecting neonates/infants (51.6%). This multicenter study highlights China’s epidemiological shift toward NAC-dominated pediatric fungemia, especially those dominated by *C. parapsilosis*, *C. tropicalis*, and emerging *C. guilliermondii*, which is accompanied by decreased azole susceptibility in NACs such as *C. tropicalis* and *C. guilliermondii*. In accordance with the WHO’s FPPL, which highlights the critical threat of antifungal resistance, these findings underscore the urgent need for enhanced antifungal stewardship and sustained national surveillance.

## MATERIALS AND METHODS

### Study design and participants

This multicenter retrospective study was conducted across 13 tertiary care children’s hospitals in China within the ISPED network, from January 2016 to December 2024. Pediatric patients with positive fungal blood cultures were included. For strain analysis, only the first positive isolate from each species per patient was included. Species were identified using standard biochemical methods. In patients with multiple episodes of candidemia, only the first episode was included. No patients had multiple positive cultures with different species during separate episodes in this study. Patients were categorized as follows: neonates (within 28 days), infants (29 days to under 1 year), and children (1 year to under 18 years). Wards were categorized as follows: NICUs, PICUs, internal medicine wards, and surgical wards. The exclusion criteria were as follows: (i) inability to identify the species, (ii) repeated isolates from the same episode, and (iii) incomplete medical records.

### Antifungal susceptibility testing

Antifungal susceptibility testing was performed using the broth microdilution MIC assays, according to CLSI M27M44S (2022) guidelines. Fluconazole, voriconazole, flucytosine, and echinocandins were evaluated according to CLSI-M60-Ed2 breakpoint values (available on the CLSI website: http://www.CLSI.org), while AMB was evaluated using EUCAST breakpoint values due to the lack of CLSI breakpoints (available on the EUCAST website: http://www.eucast.org). For species with established CLSI or EUCAST clinical breakpoints, isolates were categorized as susceptible (S), intermediate (I), or resistant (R). For species lacking CLSI/EUCAST-defined breakpoints, we applied epidemiological cutoff values (ECVs) from the CLSI M27 document. ECVs were used to categorize isolates as WT or non-wild type (Table S1).

### Statistical analysis

SPSS (v26.0, IBM Corp., USA) was used for analyses. Continuous variables had skewed distributions and are presented as medians (ranges); discrete variables are presented as numbers or percentages. Bivariate analysis for qualitative variables was carried out by the *χ*^2^ test (*n* ≥ 10) and Fisher’s exact test (*n* < 10). A Monte Carlo simulation (100,000 replicates) was used if Fisher’s exact test could not be performed. Temporal trends were assessed via the Cochran-Armitage test. The significance level was set at *P* < 0.05 with two-tailed tests.

## SUPPLEMENTAL MATERIAL

Table S1 aac.00031-26-s0001.docx Clinical breakpoints or epidemiological cutoff values for the different *Candida* species.

ASM does not own the copyrights to Supplemental Material that may be linked to, or accessed through, an article. The authors have granted ASM a non-exclusive, world-wide license to publish the Supplemental Material files. Please contact the corresponding author directly for reuse.
