# Supplementary material for: Pediatric candidemia epidemiology and antifungal susceptibility profiles reported by the ISPED program in China, 2016–2024
Source: Antimicrob Agents Chemother. 2026 Jun 2;70(7):e00031-26. doi: 10.1128/aac.00031-26 (PMC13321828; doi:10.1128/aac.00031-26)
Supplement: Table S1 — Clinical breakpoints or epidemiological cutoff values for the different Candida species. [file aac.00031-26-s0001.docx]

**SUPPLEMENTAL TABLE 1** Clinical breakpoints or epidemiological cutoff values for the different *Candida* species.

| Species | FLU  (μg/ml) | VOR  (μg/ml) | ITR  (μg/ml) | AMB  (μg/ml) | FCT*  (μg/ml) | Echinocandins  (μg/ml) |
| --- | --- | --- | --- | --- | --- | --- |
| *C. albicans* | S: ≤2^a^  SDD: 4  R: ≥8 | S: ≤0.125^a^  I: 0.25-0.5  R: ≥1 | S: ≤0.125^c^  I: 0.25-0.5  R: ≥1 | S: ≤1^b^  R: >1 | S: ≤4^e^  I: 8-16  R: ≥32 | S: ≤0.25^a^  I: 0.5  R: ≥1 |
| *C. glabrata* | SDD: ≤32 ^a^  R: ≥64 | S: ≤0.5^b*^  I: 1  R: ≥2 | WT≤0.25^d^  NWT: >0.25 | S: ≤1 ^b^  R: >1 | S: ≤4 ^c^  I: 8-16  R: ≥32 | S: ≤0.12^a^  I: 0.25  R: ≥0.5 |
| *C. parapsilosis* | S: ≤2^a^  I: 4  R: ≥8 | S: ≤0.125^a^  I: 0.25-0.5  R: ≥1 | S: ≤0.125^b^  R:>0.125 | S: ≤1 ^b^  R: >1 | S: ≤4 ^c^  I: 8-16  R: ≥32 | S: ≤2^a^  I: 4  R: ≥8 |
| *C. krusei* | NA | S: ≤0.5^a^  I: 1  R: ≥2 | WT≤0.5^d^  NWT: >0.5 | S: ≤1 ^b^  R: >1 | NA | S: ≤0.25^a^  I: 0.5  R: ≥1 |
| *C. tropicalis* | S: ≤2 ^a^  SDD: 4  R: ≥8 | S: ≤0.125^a^  I: 0.25-0.5  R: ≥1 | WT≤0.5^d^  NWT: >0.5 | S: ≤1 ^b^  R: >1 | S: ≤4 ^c^  I: 8-16  R: ≥32 | S: ≤0.25^a^  I: 0.5  R: ≥1 |
| *C. guilliermondii* | WT≤16^d^  NWT: >16 | WT≤0.25^d^  NWT: >0.25 | WT≤0.25^d^  NWT: >0.25 | WT: ≤0.5^e^  NWT: >0.5 | S: ≤4 ^c^  I: 8-16  R: ≥32 | S: ≤2^a^  I: 4  R: ≥8 |
| ^a^ Clinical Breakpoints from CLSI-M60-Ed2.  ^b^ Clinical Breakpoints from EUCAST v11.0.  ^c^ Clinical Breakpoints from CLSI- M27M44S.  ^d^ ECV from CLSI- M27M44S  ^e^ ECV from EUCAST v11.0.  *FCT should not be used as monotherapy for severe *Candida* infections  ECV, epidemiological cut-off value; I, intermediate; R, resistant; S, susceptible; SDD, susceptible-dose dependent; WT, wild type; NWT, non-wild type. | | | | | | |

MIC, minimal inhibitory concentration; FCT, flucytosine; VOR, voriconazole; ITR, itraconazole; AMB, amphotericin B; FLC, fluconazole.
